# The Methods of Choice for Extracellular Vesicles (EVs) Characterization

**DOI:** 10.3390/ijms18061153

**Published:** 2017-05-29

**Authors:** Rafał Szatanek, Monika Baj-Krzyworzeka, Jakub Zimoch, Małgorzata Lekka, Maciej Siedlar, Jarek Baran

**Affiliations:** 1 Department of Clinical Immunology, Institute of Paediatrics, Jagiellonian University Medical College, 30-663 Krakow, Poland; rafal.szatanek@uj.edu.pl (R.S.); mibaj@cyf-kr.edu.pl (M.B.-K.); misiedla@cyf-kr.edu.pl (M.S.); 2 Tissue Biology Research Unit, Department of Surgery, University Children’s Hospital Zurich, 8032 Zurich, Switzerland; jakub.zimoch@kispi.uzh.ch; 3 Department of Research of Biophysical Microstructure, Institute of Nuclear Physics Polish Academy of Sciences, 31-342 Krakow, Poland; malgorzata.lekka@gmail.com

**Keywords:** extracellular vesicles (EVs), exosomes, microvesicles (MVs), flow cytometry, dynamic light scattering (DLS), stimulated emission depletion microscopy (STED), nanoparticle tracking analysis (NTA), transmission electron microscopy (TEM), cryo-electron microscopy (Cryo-EM), atomic force microscopy (AFM)

## Abstract

In recent years, extracellular vesicles (EVs) have become a subject of intense study. These membrane-enclosed spherical structures are secreted by almost every cell type and are engaged in the transport of cellular content (cargo) from parental to target cells. The impact of EVs transfer has been observed in many vital cellular processes including cell-to-cell communication and immune response modulation; thus, a fast and precise characterization of EVs may be relevant for both scientific and diagnostic purposes. In this review, the most popular analytical techniques used in EVs studies are presented with the emphasis on exosomes and microvesicles characterization.

## 1. Extracellular Vesicles

Extracellular vesicles (EVs) are defined as membrane fragments released during the lifespan of a vast majority of cells. These vesicles are surrounded by a phospholipid bilayer, whose composition is very similar to the cell of origin. EVs carry a large repertoire of molecules including proteins (e.g., cytokines, receptors, or their ligands), nucleic acids (DNA, mRNA, and miRNA), and lipids. The lumen of EVs forms a perfect environment for biologically active components [1]. Transport in the bloodstream of signaling molecules such as hormones is not problematic, but it becomes almost impossible in the case of highly-degradable molecules such as nucleic acids. The lipid bilayer of EVs protects these molecules from degradation in the extracellular milieu, and thus allows their “safe” delivery to the target cell. For example, the delivery of miRNA by exosomes enables very fast alterations of gene expression in the targeted cells [2].

The EVs molecular composition, defined by both the inside cargo and the components present in the vesicles’ membrane, presumes their functions [3,4,5]. Although, EVs content reproduces the properties and status of the parental cell, the protein and nucleic acid composition indicates the involvement of specific, yet still unknown, mechanisms leading to their release. Initially, it was thought that the EVs release is due to disposal of superfluous or harmful content [4], however, the accumulated evidence shows that, most probably, the role of EVs is to emit signaling and regulatory molecules [4,5,6]. The specific molecular composition enables them to be recognized by, or transferred to, other cells. In that manner they can influence the phenotype and function of the recipient cell. The interaction with other cells can proceed through different mechanisms, including specific ligand-receptor interactions activating intracellular pathways or internalization by the recipient cells through membrane fusion or via endocytosis/phagocytosis with the subsequent transfer and release of EVs cargo [2,7]. With the discovery of miRNA transported by exosomes, the growing evidence links a specific miRNA pattern present in blood serum with a certain type of cancer [7,8].

**Figure 1:**
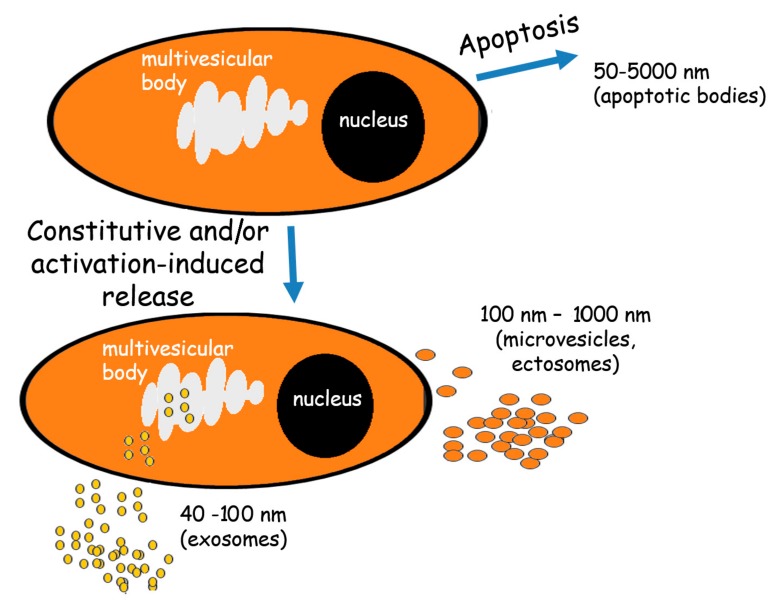
The extracellular vesicles (EVs) release. Alive cells release both exosomes and microvesicles either constitutively and/or under activation. Exosomes are formed from multivesicular bodies while microvesicles arise through direct budding from the plasma membrane. The cells undergoing apoptosis release apoptotic bodies formed by random blebbing.

The adopted EVs classification system [9] divides these membrane structures into three groups: exosomes, ectosomes (referred here as microvesicles, MVs), and apoptotic bodies (Figure 1). It should be pointed out that each individual cell is capable of releasing both exosomes and MVs simultaneously. Their presence can be detected in all body fluids (such as blood, lymph, saliva, urine, cerebrospinal fluids, breast milk, and pleural effusions of ascites) at high levels: one microliter of blood serum may contain over 3 million vesicles [10,11,12].

Exosomes have been identified to be released from multivesicular bodies during their fusion with the plasma membrane. They are small vesicles characterized by a diameter range of 40–100 nm and by a density of 1.13–1.19 g/cm^2^ [13,14,15]. They can be identified by specific markers indicating their endocytic origin, such as ALG-2-interacting protein X (Alix), tumor susceptibility gene 101 (*TSG101*), and tetraspanins [16], however, today a combination of these markers is preferred.

Microvesicles (MVs) are shed from the plasma membrane through direct outward budding of the plasma membrane, which defines their diameter and molecular composition [12,17]. The MVs size varies between 100 to 1000 nm [12,18]. They are released to the extracellular milieu after a selective incorporation of proteins, nucleic acids and lipids, and thus they are more heterogeneous than exosomes bearing surface markers such as integrins or selectins. The expression level of these markers reflects the properties of the parental cells [16,19]. Unlike exosomes, there is no specific marker defining MVs.

Apoptotic bodies are released upon cell fragmentation during late phase of apoptosis. Their diameter varies from 50 to 5000 nm [18]. This type of EVs can be identified by the detection of DNA and histones.

Since EVs are shed during cell growth, activation, proliferation, senescence, and apoptosis by different cell types, their ability to transfer functional cargo is an important factor in cell-to-cell communication, immune responses, signaling cascades, etc. [20,21,22,23]. In particular, MVs and exosomes are considered to be novel mediators of cell-to-cell communication that play an important role in both physiological and pathological processes. Therefore, it is of great interest not only to develop reliable isolation protocols but also to study their molecular, biochemical and biophysical properties [17,24,25]. The biological importance of EVs is emphasized by the fact that their cargo possesses a significant regulatory potential. Acting as conveyors of information, they can deliver their cargo to specific distant targets [10,26]. Messages convoyed by vesicles include signals crucial for cellular division, survival, differentiation, response to stress, and apoptosis [2,27]. In the last few years, many groups have investigated EVs potential as valuable biomarkers of pathological processes, including cancer [7,28,29,30].

The ability of EVs to carry information on the physiological state of the cell of origin is a driving force for the adaptation/development of various techniques enabling the detection and characterization of their molecular, biochemical, and biophysical properties. Some of these features, such as size, morphology, concentration, cellular origin and molecular composition, can be utilized for EVs characterization. The advancement of new technologies will certainly impose changes in the techniques used for EVs research, however, this will require some time. In this review, the most common and most accessible EVs research techniques are discussed, with the emphasis on exosomes and mircovesicles. Table 1 enlists the most widely used techniques in EVs studies.

## 2. Flow Cytometry and Related Methods

**Figure 2:**
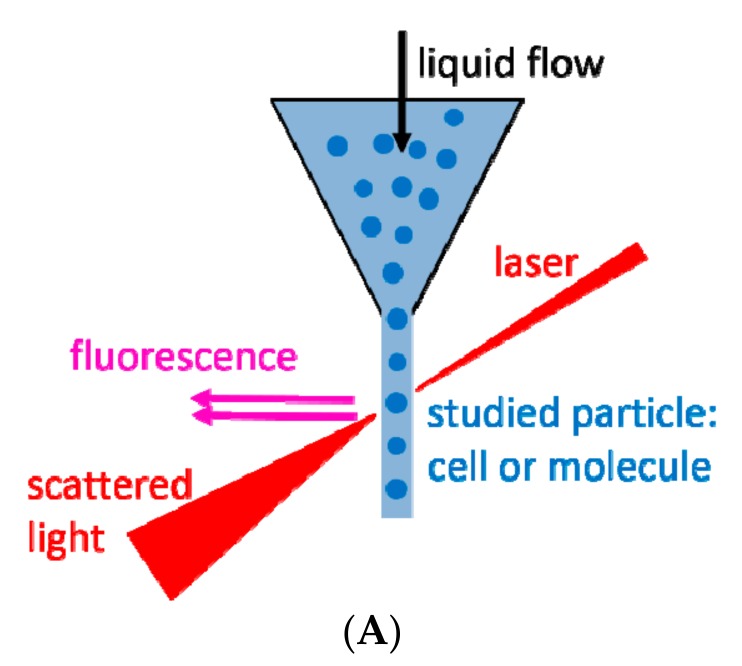

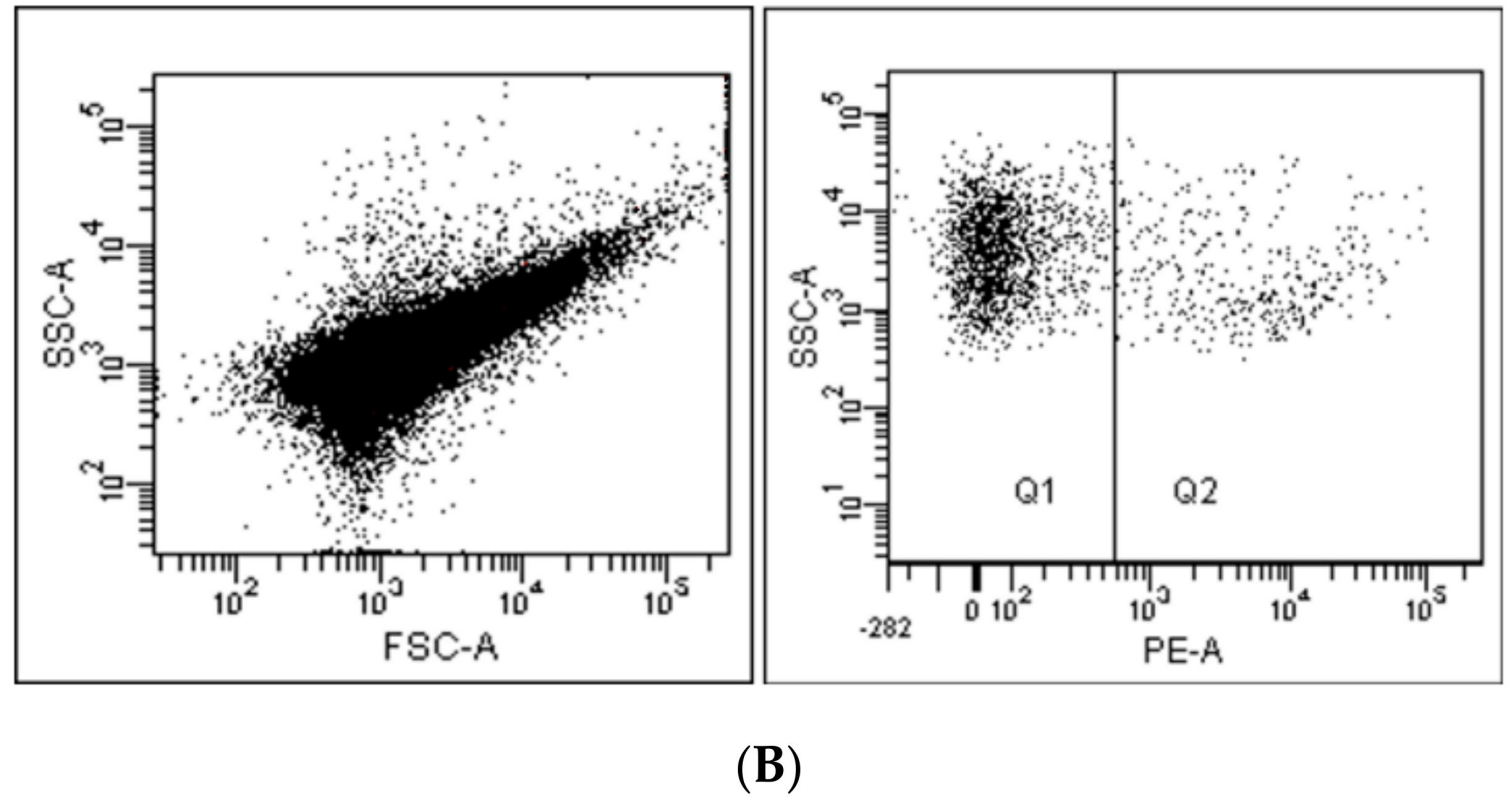
(**A**) The principal of flow cytometry; (**B**) An exemplary analysis of microvesicles (MVs) derived from HPC-4 cell line. Morphology of MVs according to forward scatter/side scatter (FSC/SSC) (**left**) and surface expression of Her-2/neu antigen detected by fluorochrome (phycoerithrin-PE) conjugated antibody (**right**). Plots from FACSCanto flow cytometer (BD Biosciences, San Jose, CA, USA).

Over the last two decades, flow cytometry has been regarded as one of the most commonly used techniques for EVs analysis, with the ability to determine the cellular origin of single EVs. Although having limitations in detecting small EVs, namely exosomes, this method enables the analysis of thousands of EVs in one sample, with a simultaneous determination of multiple markers. In this technique, a laser beam with a specific wavelength is directed through a stream of a sheath fluid containing the suspended particles. The presence of particles causes light scattering (Figure 2A). In parallel, the studied particles are labeled with fluorescent dyes that can be either introduced to their interior or immobilized on their surface, e.g., by monoclonal antibodies. Relying on the recorded parameters, flow cytometry is able to analyze the relative size and granulation of the studied particles alongside all other data that could be collected from the fluorescence staining, i.e., content of the specific, fluorescently labeled, molecules. The detection of small particles on the basis of their light scattering signal is a major challenge for conventional flow cytometers [31]. This is mainly due to the limited sensitivity and resolution of flow cytometers. Most of the current flow cytometers can detect particles larger than 500 nm, few of them with improved parameters are able to detect 200 nm particles/beads [32]. Typically, the forward scattered light (0.5–5°) can provide approximate information about the size of particles [33]. EVs below 500 nm scatter laser light in the range of electronic noise and sheath fluid alone, making it difficult to resolve particles in this size range using forward scatter (FSC) thresholding [34,35]. Moreover, forward scatter is the most variable signal between instruments of different manufacturers and its proper alignment is critical. It is affected by the refractive index mismatches between the sheath fluid and the sample, beam geometry, polarization, beam stop position and collection angle [36]. On the other hand, side-scattered light (15–150°) is often collected at the 90° angle and provides information about smaller particles or granularity of internal structures. Thus, the side scatter (SSC) has been proposed as a trigger channel [36], as most cytometers show a better detection sensitivity using SSC as a trigger rather than FSC for the analysis of small particles [36,37]. Using SSC in this manner enables reproducing the sensitivity level of 190 nm latex particles [36], however, it can still lead to problems associated with EVs resolution above the background noise [38]. A newer generation of flow cytometers uses multiple angles for FSC detection, which results in better resolution of particles [32,35]. An alternative flow cytometry approach has already been applied for the detection of EVs. It consists of triggering EVs detection on a fluorescence parameter instead of light scattering to increase the separation of the EVs signals from the background [38,39,40]. While this does help resolve the EVs from the electronic noise, it requires the use of a fluorescent marker for all EVs, e.g., a lipophilic dye such as PKH26 [19].

In addition to the fact that a significant number of particles remain undetected due to the size detection limitation of conventional flow cytometers, another problem arises when high concentrations of EVs are being analyzed, which may result in the identification of multiple vesicles as a single event. This phenomenon has been described as the “swarming” effect [41]. The underlying reason for this is the fact that multiple vesicles illuminated simultaneously by the laser beam are necessary to generate a signal above the threshold. As a result many EVs are counted as a single event signal.

Another problem related to EVs analysis by flow cytometry regards the determination of their diameter (size). Usually, the information on the EVs diameter is obtained by comparing their scatter parameters with those for the standard particles (e.g., polystyrene beads) of a known diameter that are added to the sample. It should be, however, noted that the intensity of FSC is not related directly to the particle size [33]. Also, one has to keep in mind that light scattering depends not only on the particle diameter but also on the refractive index, absorption coefficient, and particle shape properties. Thus, the properties of polystyrene beads significantly differ from those of cells (and therefore the EVs) [42]. Due to their low refractive index, lipid based vesicles scatter much less light than polystyrene beads. As a consequence, the determination of EVs diameter in relation to polystyrene beads can be burdened by substantial uncertainty.

Despite the mentioned limitations of flow cytometry, this technique possesses unquestionable advantages when applied in EVs analysis. One of them is high throughput, enabling fast measurements of EVs suspended in a fluid. Additionally, if the EVs bear some antigens at their surface, it is possible to detect them by applying fluorochrome-conjugated monoclonal antibodies. As a result, the vesicles population studied can be quantified and/or classified according to the level of antigen expression (Figure 2B) [43]. Flow cytometry can also be used to enumerate EVs by adding, as an internal standard, a known number of fluorescent latex beads (Flow-Count Fluorospheres, Beckmann-Coulter, Brea, CA, USA) or using tubes containing already predefined bead numbers (TruCount tubes, BD Biosciences, San Jose, CA, USA) [37]. For smaller particles, such as exosomes (<100 nm in diameter) some other approaches can be introduced to allow their analysis by flow cytometry. These usually involve the use of latex beads coated with monoclonal antibodies, which can bind and “pull-out” EVs expressing certain determinants. Such modification allows the detection of exosomes that were previously “invisible” for flow cytometry [44].

A related technique, based on the Wallace Coulter principle [45] is termed impedance-based flow cytometry. In this system, vesicles in electrolyte solution flow through a very narrow channel -aperture (sensing zone), where each particle displaces its own volume of electrolyte. The displaced volume increases the impedance, generating a voltage pulse where the height of each pulse is proportional to the volume of the particle. In comparison to conventional flow cytometry, this technique is not dependent on the refractive index of the particles tested. However, impedance-based flow cytometry is able to resolve only EVs that are larger than 300 nm [46,47].

Recently, a relatively new flow cytometry-based method called imaging flow cytometry was shown to allow the analysis of EVs smaller than 300 nm [48,49]. This technology combines the capabilities of conventional flow cytometry with high resolution imaging at the single-cell level. By adding imaging to flow cytometry, EVs can be clearly distinguished from beads, cellular debris and/or parental cells. Moreover, in comparison to conventional flow cytometry, imaging flow cytometry has a higher level of sensitivity for fluorescence detection of smaller particles [50]. In addition, algorithms have been developed to differentiate between aggregates and true EVs [49], enabling more precise interpretation of the data.

### 2.1. Dynamic Light Scattering

**Figure 3:**
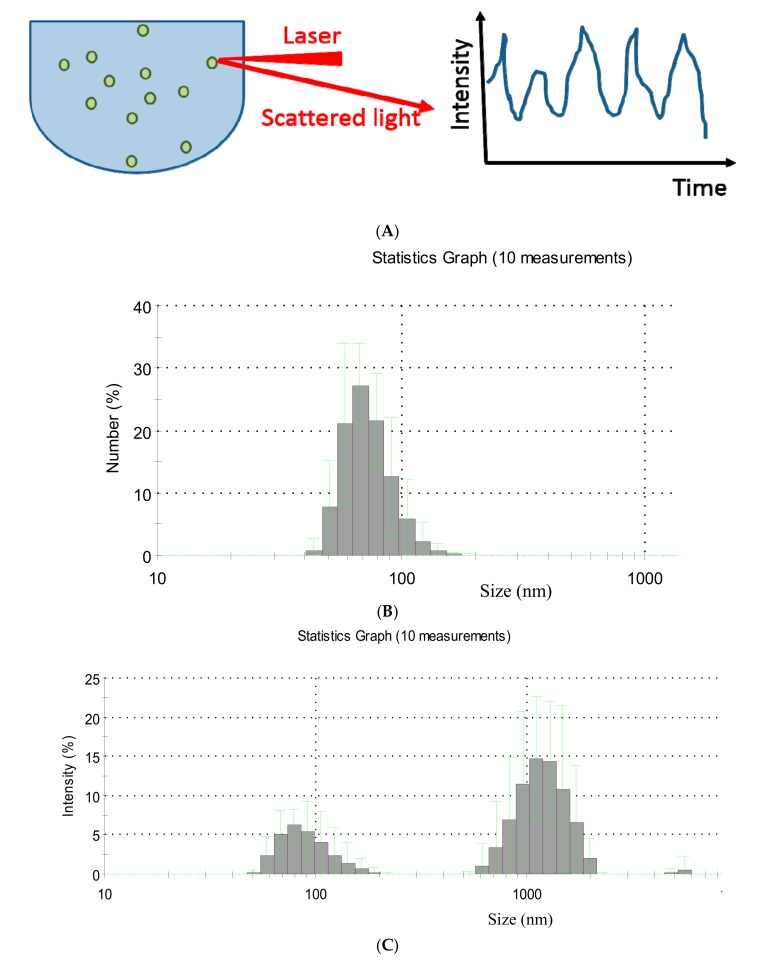
(**A**) The principle of the dynamic light scattering; (**B**,**C**) Exemplary spectra of dynamic light scattering (DLS) measurements of EVs present in human plasma of gastric cancer patient: (**B**) The most numerous EVs population in the sample; (**C**) EVs size distribution.

Dynamic light scattering (DLS), also known as photon correlation spectroscopy, is another technique which depends on the scattering of a laser beam. In this technique, a monochromatic and coherent laser beam passes through a suspension of particles (Figure 3A). If a particle happens to be in the beam’s way, the laser light is dispersed and scattered in all directions. By recording the intensity of the scattered light as a function of time, its fluctuations can be observed due to Brownian motion of suspended particles.

During Brownian fluctuations, the distance between scattered light beams constantly changes with time, leading to their interference, visible as minima (destructive interference) or maxima (enhanced interference) in the recorded spectrum. To obtain a distribution of particle size, the autocorrelation function of the intensity spectra is generated, that is further used for size determination. The analysis is relatively easy when suspended particles do not interfere with each other through collisions or electrostatic forces. Figure 3B,C shows the exemplary size distributions of EVs obtained using the DLS technique. The sample represents blood plasma collected from patients with gastric cancer (data were collected using Zetasizer Nano ZS apparatus, Malvern Instruments, Malvern, UK). The range of EVs size in the sample showed 2-mode distribution of approximately 80–110 and 800–1100 nm. The most numerous particle population had the size of around 80 nm. In the analyzed sample, the size distribution of EVs was dispersed indicating highly heterogeneous EVs population.

The biggest advantage of the DLS method is its ability to measure particles ranging from 1 nm to 6 μm. It should be stressed out, however, that DLS delivers reliable data only when one type of particles is present in the suspension (monodispersed suspensions). The method is less accurate in suspensions of particles varying in size (polydispersed suspensions). In such cases, the obtained profile of particle size is strongly influenced by larger particles, since they scatter more light. Therefore, when larger vesicles are present in the suspension, even in a low quantity, the detection of smaller events becomes problematic [51,52,53]. This implies the necessity to deplete the suspension that is being analyzed from any large contaminates. The DLS technique has been demonstrated to be a tool for assessing the distribution and size of EVs in the studies of red blood cell-derived procoagulant EVs [54] or EVs derived from ovarian cancer cells [55]. The results obtained clearly state that the DLS technique can provide the diameter range of analyzed vesicles, however, it is unable to deliver any biochemical data or information about cellular origin of EVs [55].

### 2.2. Nanoparticle Tracking Analysis

**Figure 4:**
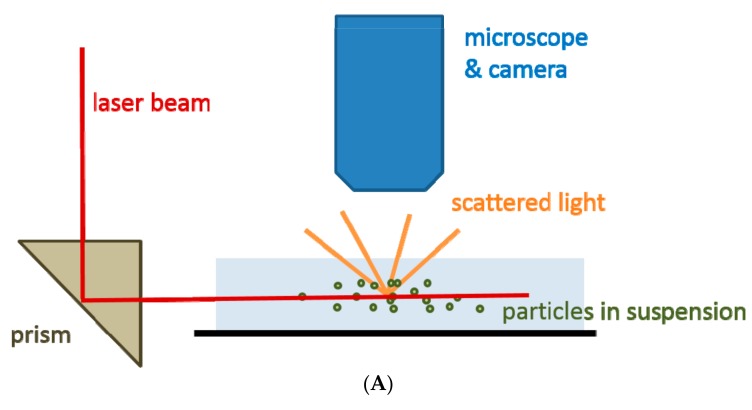

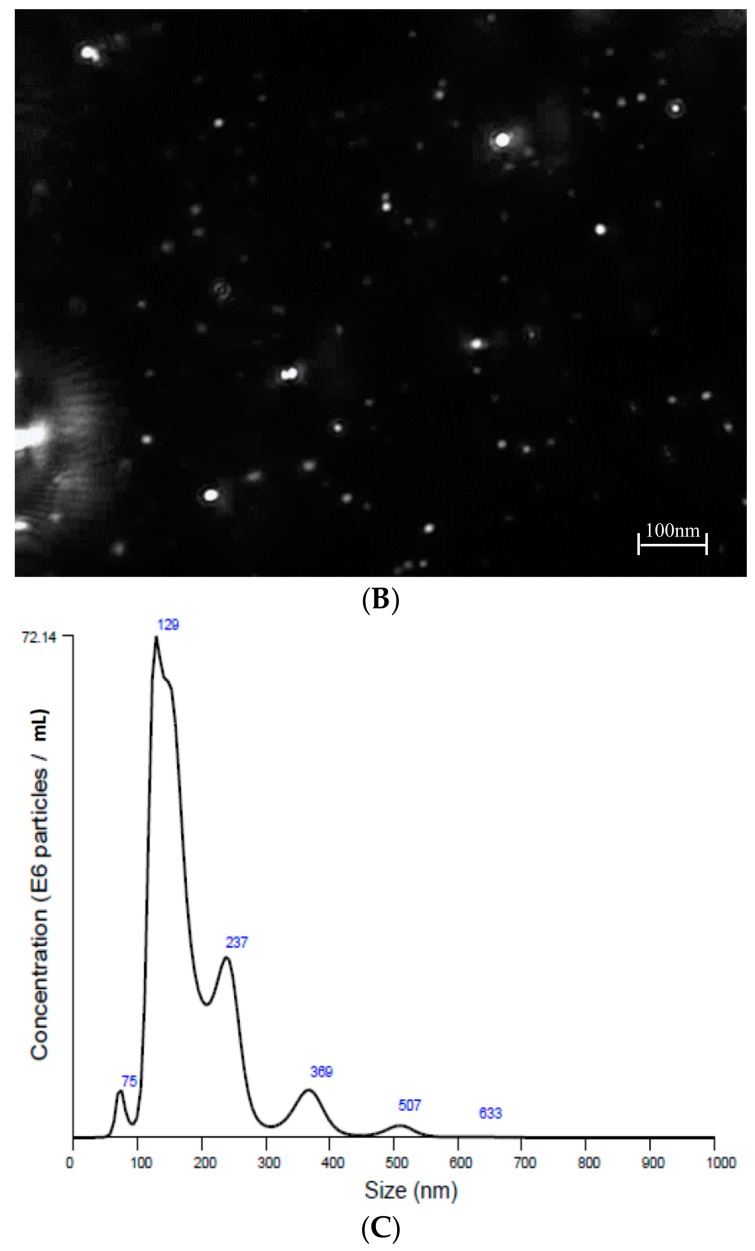
(**A**) A graphic representation of the nanoparticle tracking analysis (NTA) principle; (**B**) An image of EVs secreted by tumors cells of the gastric cancer cell line GC1401 acquired by the NTA system; (**C**) The corresponding EVs size distribution.

Nanoparticle tracking analysis (NTA), like DLS, is a technique based on the ability to track the Brownian motion of particles in suspension. The basic data about the processed particles that can be acquired by this method include average size, modal value and size distribution. The typical NTA device is composed of a laser module, a microscope connected to a sensitive charge-coupled device (CCD) or complementary metal–oxide–semiconductor (CMOS) camera, a hydraulic pump and a measuring chamber. It should be also pointed out that the measuring conditions (temperature and viscosity) should be appropriately specified in the NTA software before the actual measurement. The scheme of the NTA technique is presented in Figure 4A. The hydraulic pump puts particles present in a sample suspension into motion by injecting them into the measuring chamber at a fixed flow rate and exposing to a narrow laser beam (Figure 4B). Next, the movement of the illuminated particles over a certain time period is being recorded by a highly sensitive camera installed onto an optical microscope. From the acquired video recording, the displacement of each particle is tracked and plotted as a function of time which enables the calculation of particle size distribution by applying the two-dimensional Stokes-Einstein equation (Figure 4C).

The non-symmetric character of the size distribution data observed in Figure 4C strongly indicates that the studied suspension contains a heterogeneous population of particles, predominantly MVs (EV > 100 nm).

The use of the NTA system to detect different EVs, including exosomes, has several advantages. The first one is the ability to precisely measure small particles which diameter is as low as 30 nm. Sample acquisition is performed in a liquid phase ensuring that there are no changes to the studied EVs. Additionally, the sample preparation is very fast and easy, and the measurement itself takes only minutes. Moreover, samples can be recovered in their native form after the measurements are performed which makes this technique even more attractive.

If required, fluorescence can be also detected by the NTA system. This feature can be used for the detection of antigens present on EVs by applying fluorescently labeled antibodies. Again, the possibility of examining antigen composition as well as size distribution in smaller EVs, such as exosomes, makes this method even more appealing since other methods do not have this capability. This may be particularly important when monitoring phenotype changes in EVs present in certain diseases [56,57,58].

Alongside advantages, the NTA technique has also some limitations. One of them is a proper dilution of the sample for measurement purposes, which can be problematic especially if the sample volume is limited. The main obstacle here is to find the “right” dilution factor so that the NTA camera can register all the vesicles present in a sample and that there is no overlaying effect of a larger vesicle masking a smaller one. As with other methods based on the Brownian motion principle, the masking of smaller vesicles by larger ones, obviously, can obscure the results making them unreliable [59]. The suggested optimal particle concentration for NTA measurements is in the range of 2 × 10^8^ to 20 × 10^8^/mL [60].

Another NTA limitation concerns the detection of a fluorescent signal. Although the NTA system is capable of detecting fluorescence, its practical use for EV phenotyping is somewhat limited. Our own and others’ experience suggests that the fluorescent signal needs to be very bright in order to be detected by the NTA system in its current version [61,62]. Although fluorescent labeling with dyes (e.g., PKH27, PKH67, etc.), which incorporate into the EVs membrane, seems to be adequate for particle detection and their size/size distribution measurements, this approach does not address EVs phenotyping. On the other hand, using directly labeled fluorescent monoclonal antibodies for EVs antigen recognition by the NTA system had little success so far, especially in the case of exosomes, unless the expression of the studied marker is high (e.g., CD63) [61]. It should also be stressed that relying on fluorescence intensity as a quantification parameter may result in inadequate data acquisition. This is due to the fact that the particles move in and out of focus and the antibody flourochromes are susceptible to photobleaching. One promising alternative to solve this problem involves the use of antibodies conjugated with quantum dots (Q-dots), which are very bright fluorochromes. There are several studies reporting a successful application of Q-dot conjugated antibodies for EVs phenotyping, however, the use of these antibodies is hampered by high background coming from unbound Q-dots, which can alter the final measurements [61]. Thus, the use of fluorescent NTA for EVs phenotyping, although promising, should be treated with caution.

### 2.3. Electron Microscopy

Electron microscopy (EM) is a technique which is widely used to characterize and visualize various biological samples. It uses a beam of electrons to create an image of the studied sample. An electron beam passes through the sample where a secondary electron is generated. These electrons are collected and magnified using special lenses. In studies of biological samples, two types of EM are widely used, namely, transmission electron microscopy (TEM) and cryo-electron microscopy (cryo-EM).

**Figure 5:**
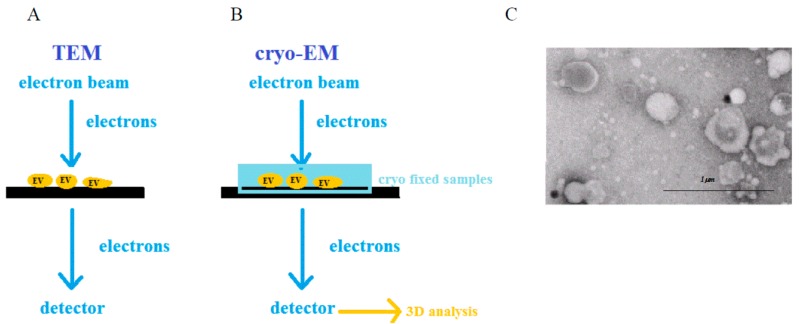
A graphic illustration of transmission electron microscopy (TEM) (**A**) and cryo-TEM (**B**) principles; TEM image of extracellular vesicles collected from plasma of gastric cancer patients (**C**).

In TEM, an image is created by electron interference when the electron beam crosses the sample. Since the wavelength of the electron beam is shorter than the wavelength of visible light by three orders of magnitude, the images are recorded with resolution of 1 nm [63]. Moreover, immuno-gold labeling opens the possibility of collecting biochemical information [64]. Unfortunately, benefits from high resolution can be easily outweighed by disadvantages related to the measurement conditions and sample preparation. The specimens analyzed by TEM have to be fixed and dehydrated before the measurement. Additionally, the image acquisition is carried out under vacuum conditions. Nonetheless, electron microscopy is utilized for EVs visualization and the obtained images are then used for diameter determination of the studied vesicles. An example of a TEM image representing a heterogeneous population of EVs isolated from the plasma of a gastric cancer patient is presented in Figure 5.

The extensive and multistep preparations needed for electron microscopy can easily induce changes in the morphology of the EVs. It has been reported by several studies that exosomes are spherical, however, other EVs are heterogeneous in shape [15]. Moreover, in some cases, the electron beam may also cause damages to biological samples. To counter these problems cryo-EM is being applied for EVs analysis, which introduces a different protocol for sample preparation. In this method, the specimen is kept and studied on vitreous ice at the temperature of liquid nitrogen, thus, the invasive steps such as dehydration or fixation are being omitted [63,65,66]. This avoids ultra-structural changes and redistribution of elements. Additionally, specimens are protected from damage caused by the electron beam’s radiation through the application of very low temperature. Due to inelastic scattering, images obtained with low-dosing techniques generate high background noise. It is possible to increase signal-to-noise ratio and retrieve higher resolution by using computer methods of single particle analysis [67]. Thus, studying biological samples with resolution lower than 1 nm is possible. Alongside two-dimensional (2-D) imaging it is also possible to generate 3-D images of specimen referred often to cryo-Electron Tomography [68]. Such 3-D imaging helped to verify spherical rather than cup-shaped morphology of exosomes, which was previously postulated based on TEM images. Furthermore, this technique allows the analysis of EVs interior. Coleman et al. has reported that exosomes are composed of more than one membrane which was confirmed by raw 2-D images as well by 3-D cryo-electron tomography [69]. As in TEM, immuno-gold staining and measurements over time can also be performed using this technique [70].

### 2.4. Atomic Force Microscopy

**Figure 6:**
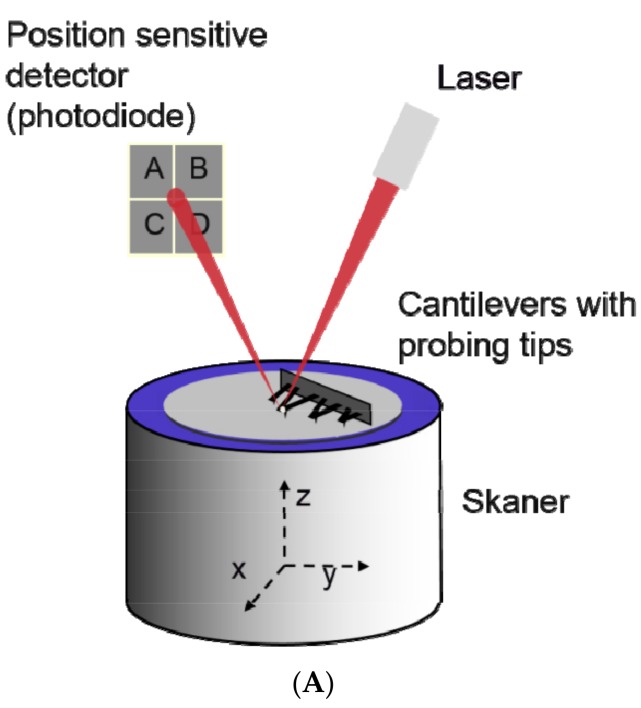

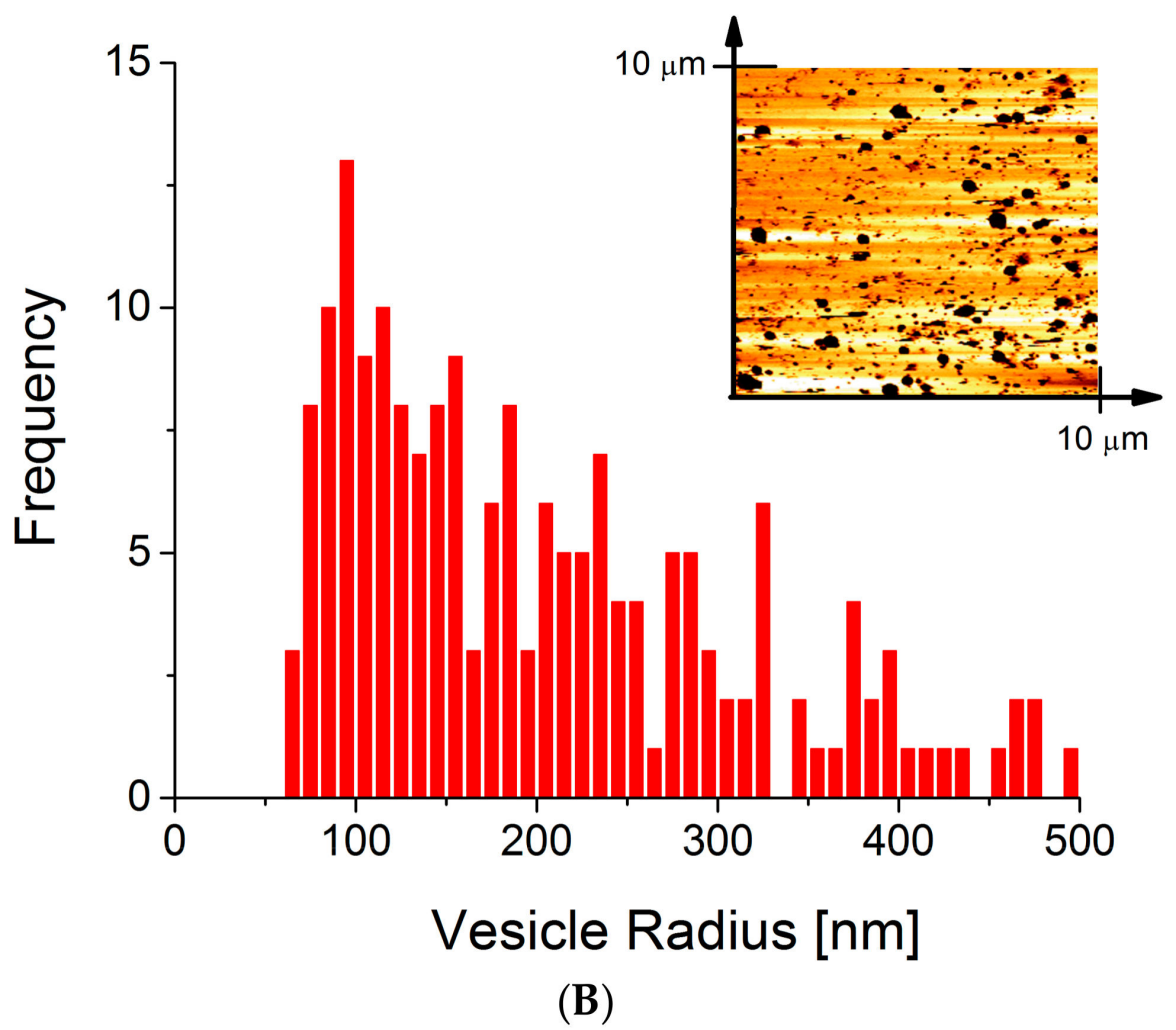
(**A**) Schematic illustration of atomic force microscopy; (**B**) The size distribution of EVs derived from HPC-4 cell line, obtained by the analysis of scanned topography image (inset).

Atomic force microscopy (AFM) is a technique, which detects and records interactions between the probing tip and the sample surface. The surface is probed by a delicate flat spring (called cantilever) with a sharp tip mounted at its free end. When the tip is brought very close to the sample, the interaction of forces leads to a deflection of the cantilever which is then recorded by the detection system comprised of a laser and a position sensitive detector (a photodiode). Figure 6A illustrates the schematic principle of AFM, while Figure 6B shows the results of EVs measurements by this technique.

An important feature of the AFM technique includes its ability to measure samples in their native conditions with minimized sample preparation [71,72,73]. For example, Figure 6B illustrates the AFM measurements of EVs obtained from HPC-4 cell lines. As required by the AFM technique, the EVs had to be first immobilized on a freshly cleaved mica surface and then scanned. The recorded topography image (Figure 6B) showed very broad distribution of the EVs. To calculate their diameter, the built-in algorithm for grain analysis was applied.

The AFM allows to obtain a real 3-D image of surface topography recorded with very high resolution, however, in order for the imaging to be successful, all vesicles must be attached to atomically flat surface such as mica. It should be mentioned that EVs can change their shape and become flattened after binding to mica surface. This can be overcome by functionalization of mica surface with molecules that can both bind to EVs and at the same time preserve the measurement conditions. Moreover, EVs bound to the surface by specific monoclonal antibodies can be used to gather quantitative information on their specific interaction with the substrate surface [72,74]. Using such an approach, it is possible to detect the presence of specific proteins with better resolution than immunogold labeling [75].

### 2.5. Other Methods

Apart of the aforementioned techniques used to characterize EVs, there are also other methods that are not utilized so frequently in such studies. These include Raman spectroscopy, resistive pulse sensing, fluorescence correlation spectroscopy, pulse laser activated cell sorter (PLACS), X-ray microscopy, stimulated emission depletion microscopy (STED) or enzyme-linked immunosorbent assays. Raman spectroscopy belongs to a group of light scattering techniques, it detects inelastic scattered light that allows interrogation and identification of vibrational states of sample molecules. As a result, it provides molecular fingerprints of the samples studied and enables monitoring of changes that occur in the molecular bond structures. Based on such measurements, the chemical composition of single EVs can be obtained [76]. Another approach uses tunable resistive pulse sensing (TRPS), which is an impedance based method (Coulter principle). In this system a voltage is applied across a size tunable nanopore filled with electrolyte, resulting in an ionic current. While passing through the pore, the particles’ resistance increases, which in turn generates a pulse that is directly proportional to the particles’ volume (www.izon.com). TRPS is used to characterize physical properties of EVs, e.g., absolute size, concentration and surface charge (ζ potential) [77]. This method eliminates limitation of an ensemble technique with intensity-weighted skewing such as DLS [78]. On the other hand, fluorescence correlation spectroscopy (FCS) is an alternative technique to DLS, that uses the analysis of fluorescence intensity fluctuations for size determination of fluorescent particles. The main advantage of FCS over DLS is its capability to detect a single, fluorescently labeled molecule. This feature drives the detection limit under 50 nm, and is less prone to erroneous results in the presence of larger particles [79,80]. Recently, Wyss et al. in an elegant study showed for the first time that this technique can be used to determine not only the average parameters such as concentration and size of the particles, but also, the number of bound antibodies (anti-CD63) per individual EVs, which corresponds to the relative expression level of a particular membrane receptor [81].

Recently, further development of existing flow cytometers resulted in pulse laser activated cell sorter (PLACS) microfluidics devices [82], enabling sorting by high speed fluid jets of individual EVs with desired fluorescence properties at the rate of ~30,000/s. These fluid jets are generated by picoliter bubbles (<100 picoliters), that are triggered by pulsed laser-induced cavitation [83] to help to eliminate swarming phenomenon.

X-ray microscopy is a relatively new imaging modality also known as micro-computed tomography (micro-CT), which can analyze samples at a resolution between that of the optical microscope and the electron microscopy. The high resolution of X-ray microscopy is achieved by reducing its wavelength to a few nm (shorter wavelength lowers the diffraction limit). X-rays experience far less scattering within samples of all types which allows thicker samples to be imaged. In the case of EVs, X-ray microscopy is a promising method for detecting the size and morphology of vesicles in their physiological state [84,85].

Stimulated emission depletion microcopy (STED) is a method that delivers high resolution images by selectively deactivating fluorophores. In this technique, the laser, tuned to the absorption spectrum of the fluorescent dye, excites the molecule into the upper electronic state. Following this first pulse an immediate second one is applied, which is red-shifted in the frequency to the emission spectrum of the dye. Such a shift in photon energy causes quenching of excited dye molecules. Whereas the first pulse is focused on the sample, the second pulse is arranged in a doughnut mode. This leads to full quenching of molecules that are present at the periphery of the doughnut. The center part remains unaffected by quenching, which leads to the increase in resolution. In this way, the STED process enables us to obtain a resolution beyond the diffraction limit [86]. The typical resolution limit for this method is around 50 nm, however, few groups reported resolution of about 10 nm [87,88]. Such measurements provide information on vesicles’ size and distribution of fluorescently labeled antigens, present at the surface of EVs [89,90].

The widely used enzyme-linked immunosorbent assays (ELISA) can be modified for the detection of EVs, especially exosomes. The kits usually consist of ELISA plates pre-coated with proprietary pan-exosome antibodies that capture exosomes from different biological samples, including cell culture supernatants and human biological fluids. Quantification and characterization of exosomal proteins is subsequently performed using appropriate detection antibodies against exosome-associated antigens whether generic or cell/tissue-specific. These kits contain also lyophilized exosomes as standards for assay calibration [91].

Very recently, Yoshioka et al. described an ultra-sensitive method, called ExoScreen, for the detection of tumor-derived exosomes in colorectal cancer patients’ serum without a purification step [92]. The method is based on an amplified luminescent proximity homogeneous assay that uses photosensitizer beads, and utilizes streptavidin-coated donor beads to capture pan-exosome specific biotinylated antibody, e.g., anti-CD63 and acceptor beads conjugated to a secondary antibody that recognizes a tissue/cell specific epitope of the exosomes. The donor beads, loaded with phtalocyanine, are excited with a laser at 680 nm, converting endogenous diatomic oxygen to singlet oxygen, which excites an amplified fluorescent signal in the acceptor beads (thioxene-loaded). As a result, the acceptor beads emit light at 615 nm, detected by the luminescence plate reader. The emission occurs only if acceptor beads are within ~200 nm of donor ones, eliminating the signal coming from larger vesicles as well as from soluble proteins. By this approach, the authors were able to detect tumor-derived exosomes in a volume as low as 5 µL of patient’s plasma [92].

## 3. Summary

With increasing interest in EVs as conveyors of many vital signals under either normal or pathological conditions, new methods are being developed, or the current ones adapted, towards reliable and fast characterization of EVs properties. Recent studies have provided information about a large variety of EVs secreted by cells in response to their state [93,94]. This is particularly important in the case of various diseases, including cancer. Depending on the interest, whether diagnostic or scientific, present and future techniques should aim at a reliable and precise EVs data acquisition. Although the current EVs analysis methods are unable to simultaneously and completely assess all the key EVs parameters (i.e., size, phenotype, morphology, etc.), they are, individually, capable of obtaining all the necessary information; with limitations.

Thus, for relatively cheap and fast acquisition of EVs phenotype, methods such as flow cytometry or Coulter’s counter should be applied. In certain cases, additional information related to the particles’ size and number can be acquired by these methods. Research, demanding more precise information on size distribution or concentration should involve more sophisticated methods such as DLS or NTA. These methods seem to be capable of multiparameter data analysis that can translate into better understanding of all EVs. Obtaining highly specific data on EVs morphology may be accomplished by electron and/or atomic force microscopies, which enable the analysis of structural and biological characteristics of EVs. A more rigorous EVs characterization (i.e., microRNA, lipid and protein analysis), however, requires the application of a combination of the above methods with the addition of the “omics” technology. With the advancement of technology in sight, the EVs analysis field has a lot of room for improvement which could unlock the full potential of EVs driven cell-to-cell communication.

## Figures and Tables

**Table 1:** The most common techniques used in the studies of the EVs.

Technique	What Is Measured	Information Acquired
Flow cytometry	Scattered and fluorescent lights	Particles’ * phenotype, absolute number and size (with limitations)
Dynamic light scattering (DLS)	Intensity of scattered light as a function of time	Particles’ size typically in the submicron scale, size distribution
Nanoparticle tracking analysis (NTA)	Scattered light	Particles’ size, size distribution, concentration, phenotype (with limitations)
Scanning and transmission electron microscopy (SEM and TEM)	Scattered electron beam	Morphology, particles’ size
Atomic force microscopy (AFM)	Interaction forces between the probing tip and surface	Particles’ three-dimensional (3D)topography, diameter

* For clarity, the word “particles” here refers to EVs.

## Abbreviations

AFM	Atomic force microscopy
DLS	Dynamic light scattering
ELISA	Enzyme-linked immunosorbent assay
EV	Extracellular vesicle
FCS	Fluorescence correlation spectroscopy
FSC	Forward scatter
MV	Microvesicle
MVB	Multivesicular body
NTA	Nanoparticle tracking analysis
SSC	Side scatter
STED	Stimulated emission depletion microscopy
TEM	Transmission electron microscopy
